# Supplementary material for: Unilateral divorce laws affect women’s welfare
Source: PLoS One. 2023 Oct 25;18(10):e0289154. doi: 10.1371/journal.pone.0289154 (PMC10599560; doi:10.1371/journal.pone.0289154)
Supplement: S1 Appendix — (PDF) [file pone.0289154.s001.pdf]

Appendix

Table 4. Difference-in-differences checks, [1]

	(1) Baseline specification	(2) No untreated states	(3) No always-treated states	(4) No never-treated states	(5) Controls	(6) Unit specific trends	(7) Group specific pre trends
Panel A: Housework							
Unilateral	-11.99***	-11.33	-12.55***	-11.61***	-14.90***	-15.17***	-14.08***
	(1.921)	(5.061)	(2.733)	(2.373)	(2.145)	(2.00e-08)	(2.87e-08)
Difference w.r.t. Baseline spec.		0.653	-0.568	0.374	-2.916	-3.179	-2.091
Difference w.r.t. Main spec.		3.048	1.827	2.769	-0.521	-0.784	0.304
Panel B: Leisure							
Unilateral	23.22***	24.82***	22.59***	23.66***	24.74***	25.01***	25.12***
	(2.575)	(1.106)	(3.360)	(3.261)	(2.597)	(3.78e-08)	(1.93e-08)
Difference w.r.t. Baseline spec.		1.602	-0.628	0.447	1.521	1.797	1.903
Difference w.r.t. Main spec.		0.791	-1.439	-0.363	0.711	0.986	1.092
Panel C: Personal Care							
Unilateral	14.97***	25.76***	14.97***	16.02***	17.26***	14.15***	17.85***
	(1.630)	(0.253)	(2.893)	(1.945)	(1.710)	(1.08e-07)	
Difference w.r.t. Baseline spec.		10.79	-0.00347	1.046	2.290	-0.818	2.880
Difference w.r.t. Main spec.		9.273	-1.518	-0.468	0.776	-2.332	1.366

Clustered standard errors at state level in parentheses
*** $p<0.01$, ** $p<0.05$, * $p<0.1$

Table 5. Difference-in-differences checks, [1]

VARIABLES	(1) Housework	(2) Leisure	(3) Personal Care
Unilateral	-15.50***	26.69***	16.28***
	(2.743)	(3.597)	(2.413)
Observations	816	816	816
Number of statefip	51	51	51
State FE	Y	Y	Y
Year FE	Y	Y	Y
State-Year trends	Y	Y	Y
Already treated vs treated DiD	-14.88	24.74	17.29
Weight	0.76	0.76	0.76
Untreated vs treated DiD	-17.45	32.85	13.08
Weight	0.24	0.24	0.24

Clustered standard errors at state level in parentheses
*** p<0.01, ** p<0.05, * p<0.1

Table 6. Difference-in-differences checks, [2]

	(1)	(2)
Panel A: Weights		
Weights	Positive	Negative
Share	54%	46%
Sum	2.87	-1.87
Panel B: Correlation of weights		
Control	t-stat	correlation
Age	0.096	0.0024
Marital status	-0.225	-0.0029

References

1. A. Goodman-Bacon (2021), "Difference-in-differences with variation in treatment timing," *Journal of Econometrics*, vol. 221, no. 1, pp. 67–91.

2. De Chaisemartin, C., & d'Haultfoeuille, X. (2020). Two-way fixed effects estimators with heterogeneous treatment effects. American Economic Review, 110(9), 2964–2996.

Table 7. Unilateral & SSM regression results: Housework, Leisure and Personal Care

	(1)	(2)	(3)	(4)	(5)	(6)
Panel A: Housework						
Unilateral	-11.26***	-12.35***	-13.45***	-12.01***	-13.27***	-14.42***
	(2.487)	(2.479)	(2.391)	(1.924)	(1.995)	(1.952)
SSM	-1.931	-2.558	-2.223	-0.732	-1.174	-1.097
	(3.260)	(3.389)	(3.439)	(2.206)	(2.260)	(2.264)
Panel B: Leisure						
Unilateral	36.48***	36.75***	37.54***	23.06***	22.93***	23.82***
	(3.416)	(3.282)	(3.325)	(2.564)	(2.631)	(2.590)
SSM	-4.181	-5.099	-4.991	-4.001	-5.742	-5.507
	(4.860)	(4.819)	(4.781)	(3.812)	(3.969)	(3.949)
Panel C: Personal Care						
Unilateral	10.89***	11.94***	12.38***	14.99***	15.74***	16.49***
	(1.832)	(1.853)	(1.829)	(1.606)	(1.615)	(1.631)
SSM	0.0260	-0.101	-0.213	0.498	0.117	0.160
	(2.828)	(2.816)	(2.875)	(3.043)	(2.980)	(3.021)
Observations	49,304	49,304	49,304	63,655	63,655	63,655
Clustered variance at State level	Y	Y	Y	Y	Y	Y
State FE	Y	Y	Y	Y	Y	Y
State Year Trends	Y	Y	Y	Y	Y	Y
Year FE	Y	Y	Y	Y	Y	Y
Cohort FE		Y	Y		Y	Y
Marital FE			Y			Y
Sample	2014	2014	2014	2018	2018	2018

Clustered standard errors at state level in parentheses
*** $p<0.01$, ** $p<0.05$, * $p<0.1$
