# Supplementary material for: Unilateral divorce laws affect women’s welfare
Source: PLoS One. 2023 Oct 25;18(10):e0289154. doi: 10.1371/journal.pone.0289154 (PMC10599560; doi:10.1371/journal.pone.0289154)
Supplement: Attachment — Submitted filename: R&R PLOS ONE referee version.docx [file pone.0289154.s002.docx]

Dear referees,

Many thanks for your thoughtful comments. I have addressed each one of them below. Please let me know if you believe I need to change the document further as you consider appropriate. Please find below a reply (in red) to each comment received by each referee on a point-by-point basis.

Best regards,

**Comments to the Author**

1. Is the manuscript technically sound, and do the data support the conclusions?

The manuscript must describe a technically sound piece of scientific research with data that supports the conclusions. Experiments must have been conducted rigorously, with appropriate controls, replication, and sample sizes. The conclusions must be drawn appropriately based on the data presented.

Reviewer #1: Yes

Reviewer #2: Partly

2. Has the statistical analysis been performed appropriately and rigorously?

Reviewer #1: Yes

Reviewer #2: I Don't Know

3. Have the authors made all data underlying the findings in their manuscript fully available?

The [PLOS Data policy](https://eur03.safelinks.protection.outlook.com/?url=http%3A%2F%2Fwww.plosone.org%2Fstatic%2Fpolicies.action%23sharing&data=05%7C01%7CRiccardo.Ciacci%40alumni.eui.eu%7C2d719c2108364213073008db41a54657%7Cd3f434ee643c409f94aa6db2f23545ce%7C0%7C0%7C638175950330431296%7CUnknown%7CTWFpbGZsb3d8eyJWIjoiMC4wLjAwMDAiLCJQIjoiV2luMzIiLCJBTiI6Ik1haWwiLCJXVCI6Mn0%3D%7C3000%7C%7C%7C&sdata=NbuRHilnv9686oe4Xxf38P%2BUUQ6Lb0qP3f6mqz0%2FAAU%3D&reserved=0) requires authors to make all data underlying the findings described in their manuscript fully available without restriction, with rare exception (please refer to the Data Availability Statement in the manuscript PDF file). The data should be provided as part of the manuscript or its supporting information, or deposited to a public repository. For example, in addition to summary statistics, the data points behind means, medians and variance measures should be available. If there are restrictions on publicly sharing data—e.g. participant privacy or use of data from a third party—those must be specified.

Reviewer #1: Yes

Reviewer #2: No

Data are attached to the current submission of the R&R version of the article.

4. Is the manuscript presented in an intelligible fashion and written in standard English?

PLOS ONE does not copyedit accepted manuscripts, so the language in submitted articles must be clear, correct, and unambiguous. Any typographical or grammatical errors should be corrected at revision, so please note any specific errors here.

Reviewer #1: Yes

Reviewer #2: Yes

5. Review Comments to the Author

Please use the space provided to explain your answers to the questions above. You may also include additional comments for the author, including concerns about dual publication, research ethics, or publication ethics. (Please upload your review as an attachment if it exceeds 20,000 characters)

Reviewer #1: The paper presents a very interesting study of effect of unilateral divorces on time-use of women from a well-being perspective. Though the time-use pattern may be subjective to many more social and economic factors, the current paper and research questions on its own also presents a intriguing and novel perspective to the literature that evaluates the impact of unilateral divorce laws on divorce rates and various outcomes.

Thanks.

Reviewer #2: General comments
1. The paper is well structured in general, with a well-written theoretical framework and research question background.

Thanks.

2. At places, I didn’t find enough explanations for your decisions in analysis and arguments.
Please let me know where this occurs and I can change them as you see fit.

3. There wasn’t enough evidence in your introduction or conclusion that you’re adding something new to this literature. It would be nice to explicitly mention that at the end of the introduction and in the conclusion, you can mention in a paragraph that, concerning the current literature, what new knowledge you are adding.

I just added this in both the introduction and the conclusion. I can change them further as you see convenient.

4. Some more robustness tests might be required to prove the reliability of your analysis. More on that in the next section.

Ok, I will perform every test you suggest to give further credibility to the study.

Specific Comments
1. The abstract didn’t carry enough information about the country of the study, the methods you used and the time period. It will be nice to mention that briefly. Also, add a sentence about unilateral divorce in the abstract, as people outside the US haven’t heard of this term before.

Many thanks for this comment that might help readers to better understand the scope and results of the paper. I have added each of the suggestions you name.

2. Maybe add a few sentences about the definition of unilateral divorce in the second paragraph of your introduction, as people outside the US haven’t heard of this term before.

Done. Please let me know if I can modify it further.


3. It would be nice to explicitly mention at the end of the introduction specifically what you’re contributing to the literature and if the outcomes you’re looking at have been looked at before. If yes, then what’d they find?

I added a new paragraph tackling this comment and a previous comment about the contribution. I can further change it as you consider appropriate.

4. Clearly specify which states are part of which panels in Table 1. It will be easier to understand with years and names of states in Panel B, C and D.

I added also already treated states to the list below Table 1. They were the only set of states I did not include since the other two categories implicitly defined those states. Yet, in this way it is much clearer for readers. Many thanks for the comment.

5. Describe briefly why do you think the states that you have chosen as control states are good controls for this period? How’re they similar to the treated states regarding population size, characteristics, and race? I see that you put state fixed effects in the model, but still I’m curious about the rationale for selecting these states as controls?

I added a whole paragraph addressing this issue before the *Empirical strategy and results* subsection. I can further edit it as you see fit.

6. The results in your table 2 is in terms of minutes that they spend additionally or less for a certain activity, right? But it will be interesting to see it in terms of the per cent change in these activities, which will be effect size related to the mean size of these activities.

I analyze this issue in page 11. Namely, I state “Using summary statistics of Panel C of Table 1, tells us that unilateral divorce laws are associated to: a decay of about 10% in time spent on housework by women, an increase of about 8% in time spent on leisure and relaxing activities by women and increment of around 3% in time spent on personal care by women.” I can change it as you consider appropriate.


7. Was the Event study conducted for all the treated states together or particularly for a state? Please mention.

All the treated states together. I clarified this issue in the new version of the article.

8. I don’t understand the rationale for your event study figures very well. Why are the CIs available for only times 1 &2, and why there is a line at t=-1? Please explain that a bit more in your figure caption or results.

As for the vertical line, it is because t=-1 is the last period before the introduction of the treatment. Only confidence intervals different from zero are presented so that the figure can be zoomed in. Whenever confidence intervals are not presented we know that they are large enough to be statistically equal to zero. In the notes of the figures it is written “This figure shows the estimated coefficients and 95% confidence intervals, for statistically significant estimates,” I can edit it further with the wording you see fit.

9. Appendix Table A.1 performs a number of checks suggested by Goodman-Bacon (2021) to address issues due to the staggered treatment timing and that we use already treated states in the control group. Can you briefly mention these suggested checks, as an average reader might not be aware of those?

Sure, I modified a paragraph in page 17 to accommodate this comment. Please let me know if you would like me to carry out further changes in this direction.

10. You can also do a decomposition analysis of your DID estimate using the “bacondecomp” package in Stata or R, which will decompose the effect size and weights contributed by each of the four comparisons that you have shown to your final estimate.

I did not use this command because, as far as I know, it cannot be used in this specific setting since it relies on the xtreg command that does not allow repeated time values within the panel as it is the case of this setting. Indeed, this setting explores data across age groups, state and years (subscripts a, s,y in the empirical specification). Hence, in each state-year cell there are multiple age groups and even different marital status. Then, xtreg cannot be used and consequently the command bacondecomp does not work either.

However, to address your comment, I have added an extra check. I collapse the data at both cohort (i.e., age) and marital status level to avoid having repeated values for each state and year. Then, I run a regression using the fixed effects of the main regression (which might be seen as a further robustness check) and for such a regression I can decompose the difference-in-differences estimator using the “bacondecomp” method. I present these results in Table A.2 and add a brand-new discussion about it. Please let me know if this solves your concerns, if not I can add any further check you see fit.

11. Finally, your conclusion doesn’t discuss the literature sufficiently and places your contribution in that literature. I would advise writing a paragraph on the current state of literature and what knowledge you added to that in your conclusion. Also, mention some limitations of your analysis regarding assumptions and generalizability of your findings.

Thanks for this comment. I have edited the last paragraph of the *Conclusion* section to accommodate these comments. I can further modify it as you consider best.
