# Supplementary material for: Unilateral divorce laws affect women’s welfare
Source: PLoS One. 2023 Oct 25;18(10):e0289154. doi: 10.1371/journal.pone.0289154 (PMC10599560; doi:10.1371/journal.pone.0289154)
Supplement: Attachment — Submitted filename: Editor response.docx [file pone.0289154.s003.docx]

Dear Editor,

I have addressed each comment and provided a reply (in red). Some of them are also addressed in the new version of the paper. Please let me know if you consider appropriate to change further any aspect of the article. Many thanks for your time and help.

Thank you so much your response. This paper is really interesting and I really appreciate the author.

Many thanks for your compliments and comments.

However, there are two major types of research designs. Primary and Secondary research designs. Which one of these have you employed? Or is it combination of both.

It is a combination of both since this paper builds a mathematical model of the problem (this framework does not rely on any data source so it would be closer to primary design) and on top of that, this paper also provides an empirical analysis using different data sources (which would clearly fall in the secondary research design category)

Sir, it's stated that this article employed a double methodology. Kindly support with references from literatures and justify the double methodology. In my opinion e.g. one could say mixed method of research design and there are lots of articles/ references to support mixed method of design, which should be stated. Similarly in this study explaining the reasons and importance of using the double methodology is paramount. I find it a little confusing, when there isn't enough justification for this. Please research and find articles, books e.tc. supporting, the same that we can relate, if not available, which I suppose should be, since that's what the article is built on. Still if not available, kindly use mixed method of research and support with available and similar literatures (the simple theoretical model built should be supported with citations, the idea must have been gotten from somewhere). This is necessary to enhance research integrity, knowledge and knowledge transfer. It's the beauty and purpose of research.

Sure, please find detailed and highlighted changes in the new version of the manuscript. To this extent I have added a footnote quoting other papers that in economics also provide both theoretical and empirical analysis in the same article and I have also added more specific citations to the assumptions of the model so that the most demanding readers might find out from where they are drawn.

Please do not hesitate to contact me if you think it would be appropriate to add more information/citations on this regard. In economics it is usual to either build a mathematical model (which in economics we call theory) or to use data to study the issue (which in economics is also known as empirics).

Secondly, kindly go through the PLOS one criteria thoroughly. You must not have published the article elsewhere, whether in part or whole or on any university server as working paper. Please retract such , with proof accordingly.For Lab, Study and Registered Report Protocols: These article types are not expected to include results but may include pilot data.

This is not a published version of the study: it differs from the current version of the article, and it is a working paper version deposited in the repository of my university. Yet, I have sent an email to remove it from the afore-mentioned repository and it was removed.
